# Correction: Predicting the number of COVID-19 infections and deaths in USA

**DOI:** 10.1186/s12992-022-00837-1

**Published:** 2022-05-03

**Authors:** Amarachukwu Felix Ebubeogu, Chamberline Ekene Ozigbu, Kholoud Maswadi, Azizi Seixas, Paulinus Ofem, Donaldson F. Conserve

**Affiliations:** 1 Department of Software Engineering, University of Malaya, 50603 Kuala Lumpur, Malaysia; 2 Department of Health Services Policy and Management, Arnold School of Public, Health, Columbia, SC 29208 USA; 3 Department of Management Information Systems, Jazan University, Jazan, 45142 Saudi Arabia; 4 Department of Psychiatry and Behavioral Sciences, The University of Miami Miller School of Medicine, Miami, FL 33136 USA; 5 Department of Software Engineering, University of Malaya, 50603 Kuala Lumpur, Malaysia; 6 Department of Prevention and Community Health, Milken Institute School of Public Health, The George Washington University, Washington, 20052 USA

**Correction to: Global Health 18, 1–10 (2022)**

**https://doi.org/10.1186/s12992-022-00827-3**

Following publication of the original article [1], the authors flagged that their article had published with affiliations 3 and 6 the wrong way around in the ‘Author details”. The affiliations have since been corrected in the published article and the correct affiliations can be seen in this erratum.
